# Sea star wasting disease demography and etiology in the brooding sea star *Leptasterias* spp.

**DOI:** 10.1371/journal.pone.0225248

**Published:** 2019-11-21

**Authors:** Noah Jaffe, Renate Eberl, Jamie Bucholz, C. Sarah Cohen

**Affiliations:** 1 Estuary and Ocean Science Center, Biology Department, San Francisco State University, San Francisco, California, United States of America; 2 Santa Rosa Junior College, Santa Rosa, California, United States of America; 3 University of Wisconsin-River Falls, River Falls, Wisconsin, United States of America; Australian Bureau of Agricultural and Resource Economics and Sciences, AUSTRALIA

## Abstract

Sea star wasting disease (SSWD) describes a suite of disease signs believed to have led to catastrophic die-offs in many asteroid species, beginning in 2013. While most studies have focused on large, easily visible sea stars with widely-dispersing larvae, less information is available on the effect of this disease outbreak on smaller sea star species, such as the six-armed sea star *Leptasterias* spp. Unlike many larger sea stars, *Leptasterias* brood non-feeding young instead of broadcast-spawning planktonic larvae. Limited dispersal and thus limited gene flow may make these sea stars more vulnerable to local selective pressures, such as disease outbreaks. Here, we examined *Leptasterias* populations at sites along the California coast and documented abundance changes coincident with recent Pacific coast SSWD in 2014. Detection of *Leptasterias* in central California declined, and *Leptasterias* were not detected at multiple sites clustered around the San Francisco Bay outflow in the most recent surveys. Additionally, we categorized disease signs in *Leptasterias* in the field and laboratory, which mirrored those seen in larger sea stars in both settings. Finally, we found that magnesium chloride (MgCl_2_) slowed the progression of physical deterioration related to SSWD when applied to sea stars in the laboratory, suggesting that MgCl_2_ may prolong the survival of diseased individuals.

## Introduction

Since 2013, sea star wasting disease (SSWD) has affected over 20 different species of sea stars on both the east [1] and west coasts of North America [2–7], and has caused the largest epizootic of marine invertebrate wildlife currently known [6]. The scale and severity of impact on multiple ecologically important species of asteroids has caused great concern about ecosystem-wide effects, leading researchers and managers to call for emergency measures to track disease effects and develop models to predict future disease outbreaks [8, 9].

Reports of SSWD pathology describe a range of gross morphological signs, including necrotic lesions, twisting rays, change in turgor, “melting” appearance, ray loss and eventual death [2, 3, 6, 10, 11]. Exact pathological impacts of SSWD may vary among individuals and species, however, highlighting the need for standardized assessment and comparison.

The causative agent or agents of this epizootic are not well understood [1, 12]. While the outbreak of the disease was originally linked to a sea star-associated densovirus (SSaDV) in *Pycnopodia helianthoides*, *Pisaster ochraceus*, and *Evasterias troschelii* [6], newer findings suggest that a single virus is not the sole causative agent of the widespread disease [12]. Multiple interacting pathogens, or perhaps a general decline in asteroid immunity, possibly due to environmental stressors, may contribute to the severity and extent of SSWD [7, 12,13]. Increased sea surface temperature has been correlated with increased severity of SSWD at local and regional scales [2, 10, 11]; however, lower temperatures have been implicated as well [3]. Across larger spatial scales, anomalously high water temperature was implicated in severity of SSWD, but not outbreak itself [7]; thus, the role of temperature in SSWD is ultimately uncertain.

Sea stars such as *P*. *ochraceus* and *P*. *helianthoides* have been the focal point of most studies of SSWD thus far [2–7, 10, 14–16] due to their large size, their known ecological importance [17, 18], and the dramatic impacts of SSWD on their populations. Considerably less is known about wasting in smaller asteroids found along the North American Pacific coast, such as *Leptasterias* spp., though these species are also hypothesized to play a significant role in intertidal food webs [19–22]. In addition to its smaller size, *Leptasterias* contrasts with *P*. *ochraceus* and *P*. *helianthoides* in life history. While the latter disperse via planktonic larvae [23], *Leptasterias* lack a planktonic larval stage and brood young until they are fully developed [24–26]. This life history limits larval dispersal, which may increase vulnerability to disease events by preventing immigration and genetically isolating local populations [27–31]. Further, severe but localized mortality has been documented in *Leptasterias* in California prior to the emergence of SSWD [32, 33], highlighting the need for broad-scale surveys of regional population abundance changes, such as those occurring concurrent with SSWD [32, 34]. Size and life history are critical factors that should be compared among species when describing and reporting a disease outbreak.

The severity and widespread impact of this disease (both in space and time) mandate the establishment of pre-disease baselines making considered use of existing data from multiple sources (e.g. scientific or professional broadscale monitoring data, citizen science data, species-targeted collection data for varied purposes) to determine population level impacts. Species-targeted baseline population surveys or collections are especially important for relatively cryptic invertebrate species, such as *Leptasterias*, that are not currently the detailed focus of large-scale efforts (though see [35]). *Leptasterias* spp. are cryptic in that they are both difficult to locate in the field and to differentiate into species using morphological characters [25]. Multi-year data sets of sea star abundance have historically been rare (though see [7, 35–38]) especially those covering large geographic scales [39–41]. What datasets do exist often vary in methodology to best fit individual research objectives of smaller studies [42]; though, the coordinated ecosystem-wide monitoring in Miner et al. [35] is an outstanding example of a large, multidimensional dataset. Large-scale monitoring efforts (e.g. [7, 35]) by professional scientists, as well as those involving citizen scientists (e.g. [35, 43]), have focused on easily quantifiable, generally larger species to gain robust data across many collectors and locations. For example, while *P*. *ochraceus* is a target species in coastwide long-term monitoring surveys [35], *Leptasterias* is not.Thus, there is a need for baseline datasets for more challenging taxa with alternative life-history features that may drive different kinds of population trajectories, such as patchy distributions, highly varied abundances, and cryptic habitats [42, 44–46].

Assessment of disease condition in affected *Leptasterias* individuals can be challenging, as their small size and variable, mottled coloration complicates identification of disease signs. Thus, standardized documentation of SSWD morphological signs in *Leptasterias* is necessary to understand the range and impact of the disease as in other species [8, 47–49].

The goals of this study are to document the population-level impact and etiology of a severe mortality event in *Leptasterias*. Our primary demographic focus was detecting abundance changes in populations near the San Francisco Bay outflow in central California. To provide geographic context for these surveys and maximize coverage, we made use of existing data of varied types and goals (timed counts, transects, and quadrats; *Leptasterias*-focused and general surveys). Though we recognize potential issues with using multiple data sources, we nevertheless feel that the goals of this project are best served by including as much data as possible with appropriate caution. In addition to taking advantage of survey data, we documented morphological signs of disease as they appeared in *Leptasterias* in field and laboratory settings. We compiled these morphological signs into a 0–4 scale of disease progression with the following goals: 1) to assess the severity of infection, 2) to compare to other published infection observations (e.g., [2, 3, 11]) and 3) to allow for standardization and reproducibility across researchers examining SSWD in *Leptasterias*. Additionally, we show that magnesium chloride (MgCl_2_) application may slow progression of SSWD in a laboratory environment.

## Methods

### *Leptasterias* abundance

We used four different data sources available for sites in California: 1) timed counts (S1 Table “Cohen TC”), 2) *Leptasterias*-specific data from other projects that did not specifically assess abundance via timed counts (S1 Table “Cohen other”), 3) published *Leptasterias* abundance data [32] (S1 Table “Jurgens et al.”) and 4) data from ongoing long-term rocky intertidal monitoring focused mainly on *P*. *ochraceus* [35] (S1 Table “MARINe”). 1) and 2) were long-term surveys focused on central California sites near San Francisco Bay, sites largely excluded by 3) and 4). Shortly after SSWD was observed, 1) and 2) were expanded northward, further north than sites included in 3) but overlapping with long-term monitoring in 4) at some sites. Though the focus of 4) was not exclusively on assessment of *Leptasterias* abundance change, we sought to use as much available data as possible to give a more complete picture of abundance and provide context for San Francisco Bay outflow-associated population surveys. To account for varied sampling methods (long-term monitoring program data [32, 35], timed counts, quadrat mapping, and sample logs), we converted the highest reported *Leptasterias* total count on any single sampling date for each site and year (S1 Table “*Leptasterias* counted”) into one of five abundance ranks (S1 Table “abundance rank”). If in a given year multiple data records were available for a site, we selected the highest reported *Leptasterias* count for that year to ensure that presence of *Leptasterias* at a site was documented. Thus, the overall report is biased towards higher abundances. An abundance rank of zero indicates that *Leptasterias* were not observed in the field at that time. For sites where no *Leptasterias* were found, we report the total search effort in observer-hours as well as the total time searched in observer hours (S1 Table “*Leptasterias* counted”).

For timed searches that we carried out ourselves, sites were selected based on habitat type, geographical representation, and availability of previous records of *Leptasterias*. At each site, large areas of rocky intertidal habitat suitable for *Leptasterias* (e.g. pools, crevices, cobble, boulders, mussel beds, rocky shelves and walls) along the mid-low intertidal zone were located and GPS waypoints or physical markers were noted. Within a given time period, habitats were searched and the number of *Leptasterias* recorded. To account for the low number of *Leptasterias* at some sites, search effort of Cohen Lab timed counts is recorded as observer-hours (S1 Table “*Leptasterias*/observer hour”), the number of hours spent searching by the number of people (e.g. 3 people for 10 min represents a search effort of 0.5 observer-hours). If multiple timed searches were conducted at a single site during a single outing, the total number of *Leptasterias* from all timed searches on that date was recorded and all timed searches were used to calculate a total effort. We considered a total time of 3 hours or more solely dedicated to finding *Leptasterias* (as in the Cohen TC data) to be a robust data point, given the time and effort requirements of searching for *Leptasterias*. Counts were performed by at least 2–3 practiced observers of intertidal habitats for all central and most northern California sites and, following an initial visit, always included at least one person familiar with individual features of the site and with past observations of *Leptasterias* at that site.

While the Cohen lab has been studying *Leptasterias* at various sites in central California since 2007, projects conducted prior to the onset of SSWD were primarily focused on reproductive ecology, behavior, and population genetics. Where timed count data were not available (especially for years prior to 2014), we included available data such as number of *Leptasterias* collected for genetics and culture lab experiments or followed over time in marked plots (S1 Table “Cohen other”) at selected sites, e.g., Mussel Rock. For lack of other more specific data, we have used the conservative number of individuals collected or monitored, though in most cases the abundance is likely higher, due to time limitations of collecting trips with numerous goals.

For data from [32], we used the published total number of *Leptasterias* counted in 0.25m^2^ quadrats (S1 Table “Jurgens et al”). Data from [35] (downloaded from https://www.eeb.ucsc.edu/pacificrockyintertidal/methods/index.html; S1 Table “MARINe”) consists of annually to biannually sampled sites. We used data from all sites along the California coast that had data for *Leptasterias*. *Leptasterias* counts from 2–3 plots reported for a sampling day were added together and reported as total counts.

**Fig 1:**
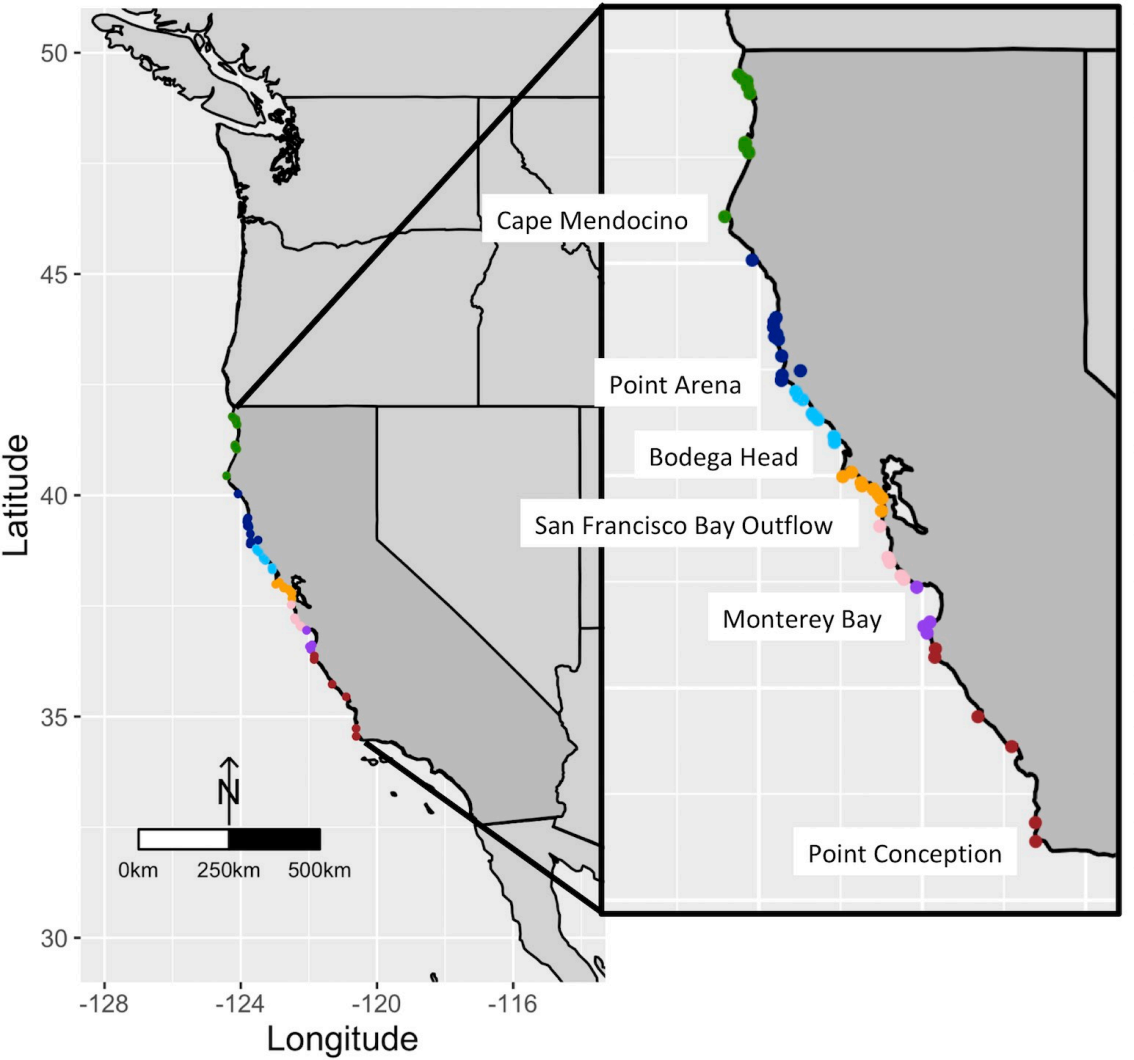
Map of abundance survey sites in California. Colored symbols indicate regional sampling areas. Refer to S1 Table for more detail.

Surveys from varied data sources ranged geographically from Del Norte County (41°47’N) to San Luis Obispo County (34°33’N; Fig 1), and spanned a time period of six years (2010–2016). Where additional data were available from select central California sites for an earlier time period (2002–2009), the highest abundance found in that entire seven year period is reported. Maps of sampling locations were created in R using the ggmap package [50].

### Field pathology observations and assessment

We observed morphological disease signs in *Leptasterias* in the field during repeated trips to ten intertidal locations between June of 2016 and June of 2017 (see S2 Table for complete list of sites). These sites range geographically from Friday Harbor, Washington (48°32’N) to San Mateo County, CA (37°11’N) and are varied topographically, often characterized by large boulders or cobble extending into the ocean, shallow channels, and pools. These tidal areas are exposed to varying degrees of wave impact, and support a diversity of algae and sessile invertebrates. *Leptasterias* often reside on exposed rock faces, in crevices, in mussel beds, or in or near pools, often in contact with algae or seagrass.

To assess SSWD severity, a 0–4 scale for *Leptasterias* was developed (Table 1) based on previous work on SSWD in *P*. *ochraceus* [2,3, 10, 35] and signs of wasting observed in *Leptasterias* in the field and lab.

**Table 1:** SSWD morphology categories.

Numeric Category	Category Title	Observable morphological signs
**0**	Visually unafflicted.	Unable to detect visual signs of SSWD.
**1**	Potentially afflicted but not certain.	Single, small lesion (<¼ surface area of arm), though may not have lesions at this stage; mild abrasion at end of rays; soft and spongy tissue; white; “milky” tissue, though may be difficult to distinguish between normal mottling coloration of *Leptasterias* spp.
**2**	Definite morphological damage related to SSWD.	Single or numerous small lesions (approximately ¼ surface area of one or more arms); limited ossicle exposure; some arm tips show mutilation; mild deflation, twisting rays; rays beginning to detach from body.
**3**	Severe morphological damage related to SSWD.	Lesions on two or more arms (more than ¼ surface area of arm) or single extensive lesion (more than ¾ surface area of arm); tissue disruption sufficient to allow clear ossicle exposure; severe deflation; loss of one or more rays.
**4**	Very severe morphological damage related to SSWD.	Many large lesions (lesions on two or more arms, tissue disruption on arms takes up more than ½ of surface area on arm); collapse of body wall in areas and exposure of much of ossicle surface; loss of several (two or more) rays.

0–4 scale of morphological signs associated with SSWD at each stage.SSWD pathological impacts at each stage may include some or all of these signs.

Sea stars were located and collected in small plastic containers for individual assessment using the 0–4 scale (see above). Individual sea stars were scrutinized in great detail, generally by multiple observers, who compared assessments so that a consensus score could be recorded and a common methodology developed. Photographs were also taken of sea stars submerged in water in plastic tubs, and, when possible, sea stars were further assessed using dissecting microscopes soon after collection in the field, so that field and lab microscopy assessments could be compared. Particular care was taken to consider damage related to SSWD as distinct from other types of damage common to intertidal sea stars [51].

### Laboratory pathology observations

Both visually healthy and visually diseased *Leptasterias* individuals were collected from three sites in northern California (Point St. George, Battery Point, and Belinda Point) and two sites in Oregon (Fogarty Creek, Boiler Bay; S1 Table), and kept in individual aquaria (~14°C, ~3.0% salinity; water quality monitored daily).

During the experimental treatment period (6/20/16–8/8/16), sea stars were photographed daily with a Canon EOS T2i Rebel camera and a Nikon SMZ800 dissecting microscope. Photographs were taken beginning at the time initial signs of wasting were observed and ending either at sea star death or the end of the treatment period. Sea stars were removed from tanks for photography and placed in 80 mL of seawater (3.0–3.2% salinity) in a 237 mL glass dish. Two relaxation treatments were given to sea stars to slow down motor function for photography.

All sea stars were chilled on ice for 5–20 minutes each day prior to photography, following a protocol for relaxing marine invertebrates [52]. The treatment group of sea stars received 20 mL of 0.37M MgCl_2_ added to 80 mL of seawater, and were placed in a refrigerator at ~7°C for 5 minutes. Temperatures were recorded before sea stars were removed from tanks, after they remained in bowls on ice for five minutes, after removal from the refrigerator, and again before returning them to the tank. Careful precautions were taken to avoid cross-contamination, e.g. hands were washed between each sea star and dishes were bleach-treated and left to dry before reuse. The number of total days of observations varied among sea stars because not all sea stars displayed pathological signs of SSWD at the same time during the fixed dates of the experimental period.

A Logrank test was conducted in Statistix 10 [53] to assess the effect of the MgCl_2_ treatment on sea star disease progression and longevity following observation of early signs of wasting disease.

## Results

### *Leptasterias* abundance

**Fig 2:**
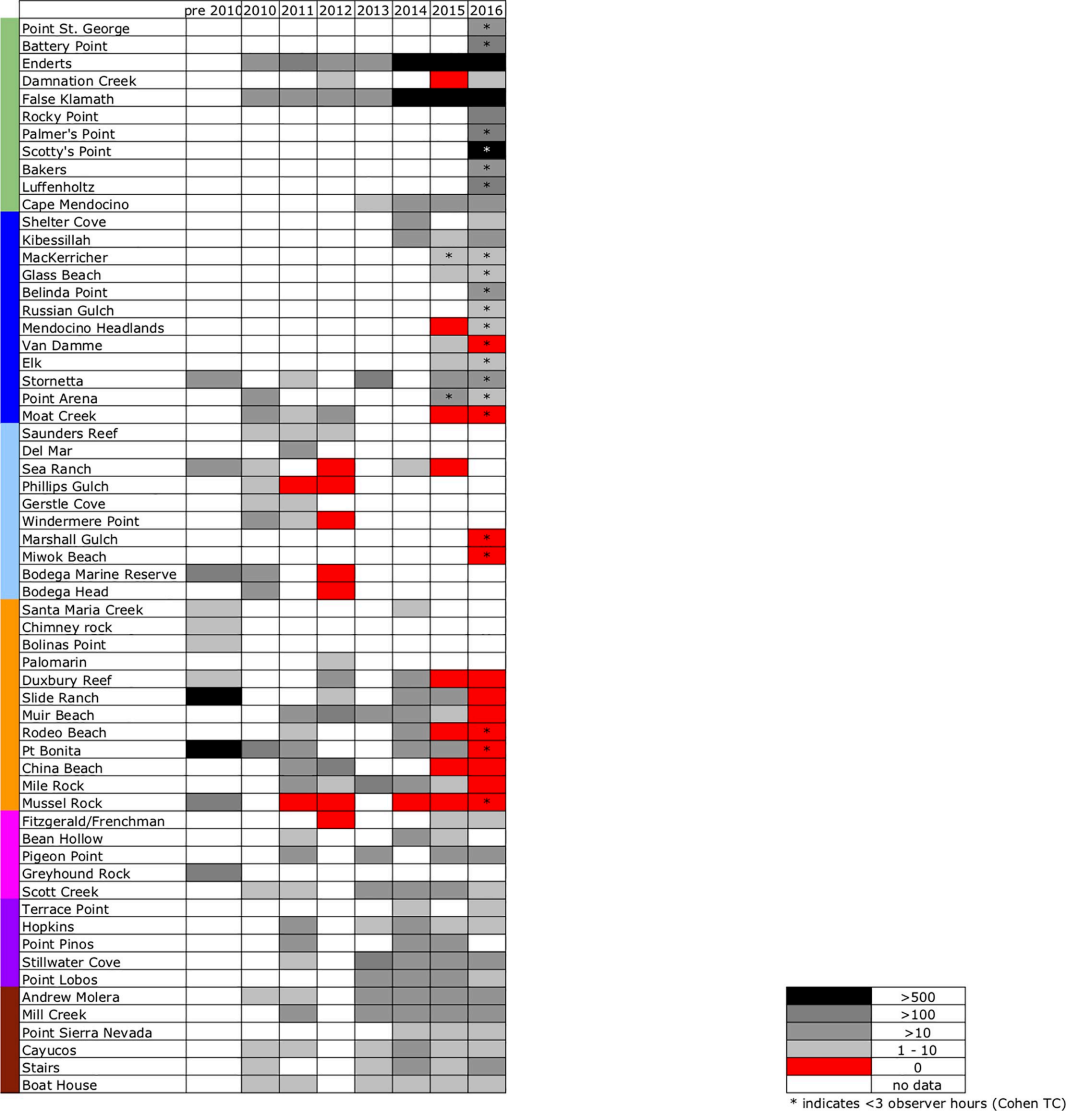
*Leptasterias* abundance observed from 2010 to 2016. Sampling sites from North to South (41°47’N to 36°38’N). Each square represents the greatest number of *Leptasterias* found per sampling day for a particular year converted to abundance ranking as follows: black—abundant (>500 individuals); dark grey—common (100–499 individuals); medium grey—present (10–99 individuals); light grey—rare (1–9 individuals); red—no *Leptasterias*; white—no data. Asterisks indicate observations ≤3 observer hours.

The data set consisting of 46 sites along the California coast from 41°47’N to 34°33’N was divided into seven regions based on geographical or oceanographic features (Fig 1, colored points; [54, 55]). We report regional differences in detection in 2015–2016, with a notable absence of *Leptasterias observ*ations in at least five sites in a region associated with the San Francisco Bay outflow, spanning approximately 35 km of outer coastline extending both north and south of the Golden Gate Bridge (Fig 1 orange points; Fig 2; source data, as in Sup file: Cohen TC, Cohen other, MARINe).

We further report that during this period, both our timed counts and data from [35] showed abundances in the 100s at multiple sites north of Cape Mendocino, including Enderts, False Klamath, and Rocky Point. In contrast, *Leptasterias* were observed in much lower abundance at sites south of San Francisco Bay, where many sites showed abundance in the 10s and below with the survey methods employed (source for southern data: MARINe. Source for Northern data: MARINe; Cohen TC). (Note: due to low volume of past and recent surveys at some sites, differences between the “zero” and “1–10” ranks may not reflect actual differences in abundance, and be caused instead by limited sampling). The highest reported abundance at these southern sites in 2016 was at Pigeon Point, where 60 *Leptasterias* individuals were observed.

### Detailed observation of pathology in the field

**Fig 3:**
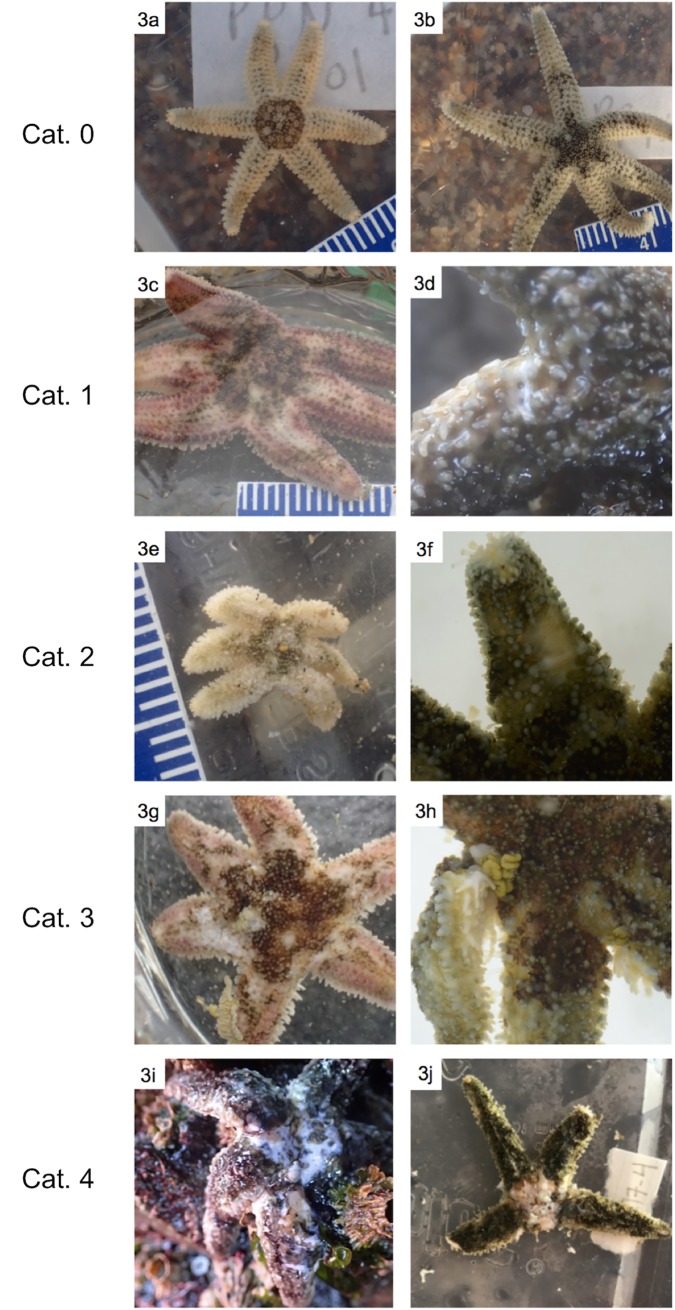
Examples of gross morphological signs observed in field *Leptasterias* across stages of SSWD (categories 0–4). (a) and (b): Visually healthy *Leptasterias*—category *0*. (c) Possible lesions on multiple arms; however, may be mottled coloration—category *1*. (d) Axillary lesion, possibly the result of SSWD, magnified to show ossicle exposure—category *1*. (e) Deflated appearance, lesions on multiple arms—category *2*. (f) Distal lesion, possible abrasion at arm tip—category *2*. (g) Deflated appearance, body wall rupture in central disk and arms, internal organs emerging from disc and arm—category *3*. (h) Axillary body wall rupture, advanced arm autotomization—category *3*. (i) Extremely deflated appearance, necrosis in majority of central disk, body wall rupture across most of body—category *4*. (j) Body wall rupture across majority of central disk, complete autotomization of multiple arms, severe abrasion on multiple arm tips—category *4*.

*Leptasterias* assessed for wasting in the field showed SSWD morphological signs ranging from completely absent to extremely severe, including several *Leptasterias* that were presumed dead, showing disintegration across much of the body wall and lack of movement of tube feet. Individuals were assessed using a 0–4 wasting scale (Table 1). Photographs of *Leptasterias* individuals have been included to show examples of each stage (Fig 3).

### Lab pathology observations

**Fig 4:**
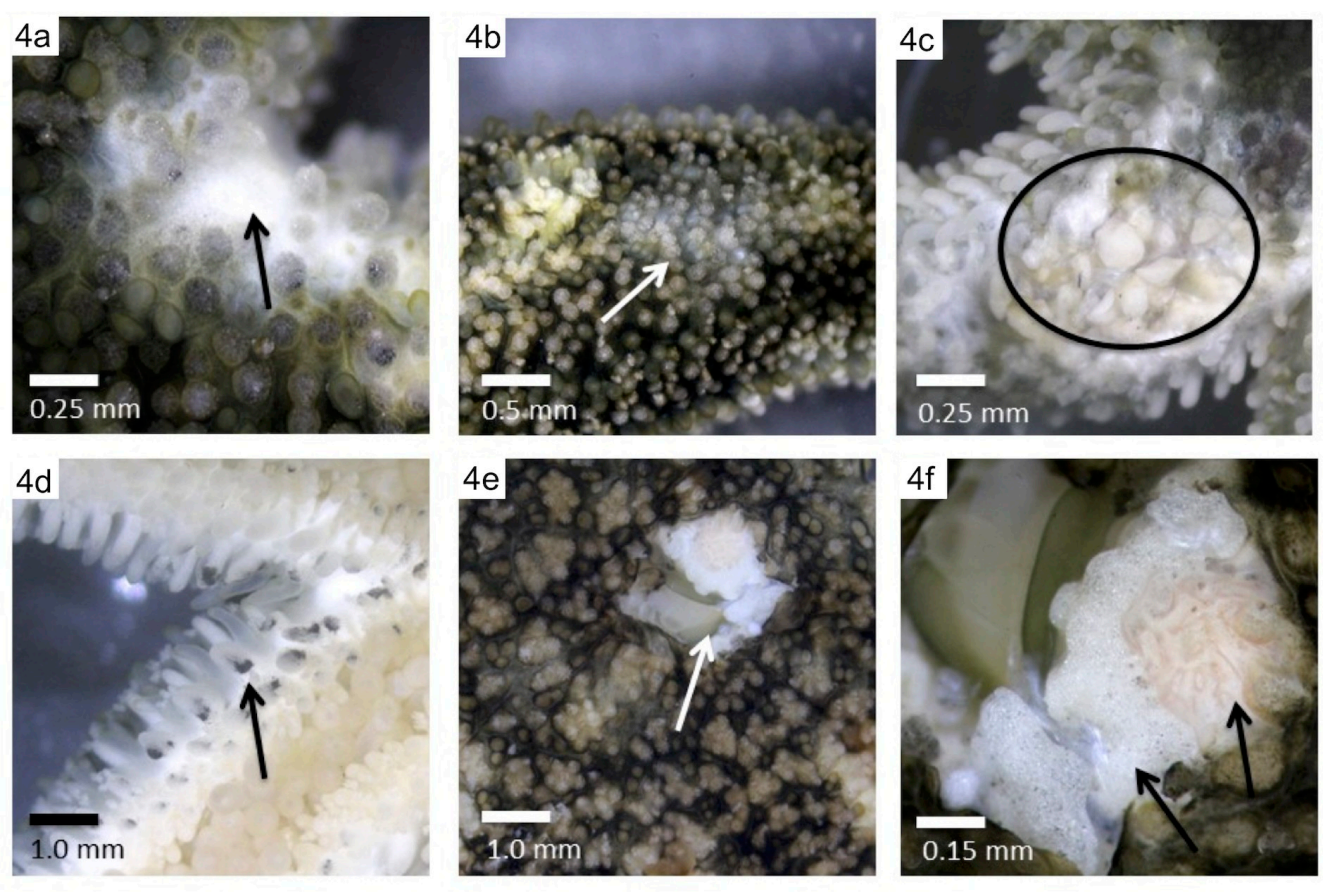
Morphological signs of disease observed in the laboratory. (a) Wasting lesion in axilla. (b) Early signs of wasting: white lesion on ray. (c) Wasting lesion on ray with ossicle exposure. (d) Black tissue on tube feet. (e) Wasting lesion on central disk. (f) Close-up of lesion seen in *5e* with arrows denoting madreporite and exposed ossicles.

**Fig 5:**
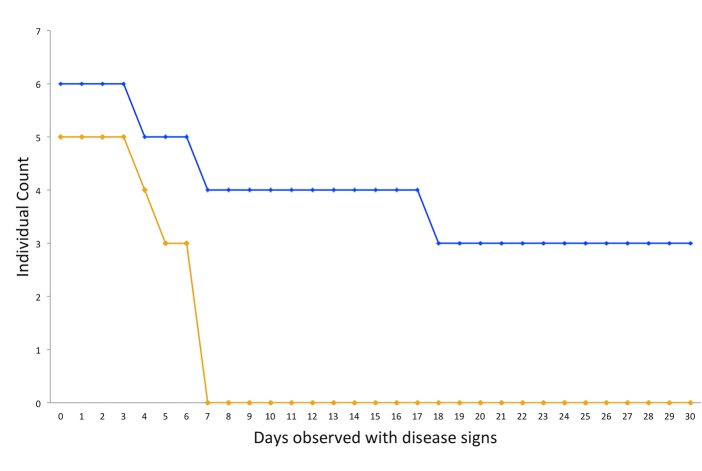
Survivorship plot depicting the number of days from initial signs of wasting until death or end of experiment, with MgCl_2_ + ice treatment in blue, and ice-only treatment in orange.

A range of pathological signs was observed in *Leptasterias* individuals (n = 11) that were kept in the lab for the duration of the experiment. The most commonly observed pathological signs were lesions on the axilla (n = 4 individuals; Fig 4A); lesions on the rays (n = 3 individuals; Fig 4B and 4C); necrosis on proximal pedicellariae (n = 1 individual; Fig 4D); and a hole on the central disk (n = 3 individuals; Fig 4E and 4F). Wasting progression to death in the ice-only group was an average of 6 days (range 3–7 days), while wasting progression to death or the end of observation period in the MgCl_2_ and ice group was on average 22.1 days (range 3–46 days; Fig 5). Three *Leptasterias* lived for the duration of the experiment in the MgCl_2_ and ice group (30 days; Fig 5). Neither disease progression nor regression was observed in the sea stars that survived. A Logrank test in Statistix 10 [53] was not statistically significant (p-value = 0.07), perhaps related to the small sample size of the treatment groups.

## Discussion

Findings of low abundance in *Leptasterias* varied locally but were most severe in central California, including multiple populations around the San Francisco Bay outflow where *Leptasterias* was not observed in the most recent surveys (west of the Golden Gate Bridge, outside the bay; Fig 1, orange points). In contrast, populations north of Cape Mendocino (Fig 1, green points) were observed after the SSWD outbreak, and showed high levels of abundance in most recent surveys (Fig 2), despite the observation of many sea stars in this region displaying signs of disease. (Note: *Leptasterias* in Sonoma County (Fig 1, light blue points) had previously showed population declines and functional extirpations associated with a harmful algal bloom [32].) Results of these surveys show that disease impact was variable and may be affected by multiple factors. Gross features of field pathology matched that in the laboratory and etiologies described in other species [2, 3, 6, 10, 11].

### Possible factors contributing to decrease in detection of *Leptasterias*

SSWD outbreaks and associated population declines are likely influenced by a multitude of factors. Classically, higher host population density is thought to increase disease transmission (e.g., [56, 57]) and was correlated with SSWD in *P*. *ochraceus* on a local scale [2], but not broadly [7]. Population density has also been implicated as a contributing factor in a recent mortality event observed in the circum-antarctic sea star *Odontaster validus* [58], though this event differed from SSWD in etiology and is likely unrelated. In *Leptasterias*, some sites where pre-outbreak observations often showed high densities of individuals, such as Slide Ranch, no longer show evidence of such high density after SSWD appeared in the two following years (Fig 2; S1 Table). Although the connection between population density and SSWD incidence is not clear (Miner et al. [7] found no such link), density is one factor among many that researchers should continue to monitor and compare to gain a holistic understanding of disease outbreaks such as SSWD.

Given that SSWD has been reported in up to twenty species of asteroids [2, 6], cross-species infection may be possible, even if the impact of the disease varies greatly among species [35]. Any reduction in host populations would reduce contact between infected and uninfected sea stars, which could reduce the chance of density-dependent transmission [59]. This holds true for changes in overall sea star density as well as species-specific density changes, so it is possible that an initial severe population decline in some of the larger species may have decreased the cross-species contact rate and lowered the risk of infection of *Leptasterias*.

*Leptasterias* population genetic composition across sites may affect disease impact and may be linked to differential mortality. *Leptasterias* have limited dispersal potential at all life history stages [24–26] and thus may be prone to local adaptation [27, 55]. High levels of differentiation across sites could lead to dramatically different susceptibilities among clades [60]. Recent fine-scale molecular analysis of Central California *Leptasterias* [61, 62] revealed a “Bay-proximal” clade found almost exclusively at sites near San Francisco Bay where no *Leptasterias* were observed in 2015–2016 (Fig 2). Differences in observed abundance at sites with different clade composition [61, 62] suggest that differential susceptibility may exist among different *Leptasterias* clades. Further, significant allele frequency changes over time were associated with SSWD in *P*. *ochraceus* [63], suggesting that genetic differences in susceptibility to SSWD could lead to differential survivorship and selection.

Local abiotic effects may also affect disease impact. Drastic changes in abundance in echinoderms have been associated with direct and indirect anthropogenic effects [64]. San Francisco Bay represents one of the largest urban centers along the west coast of the United States and the largest population center within the geographic range of this study. Hence, *Leptasterias* in coastal sites near the San Francisco Bay outflow (e.g., Mile Rock, Muir Beach, Pt. Bonita) may experience multiple anthropogenic stressors, such as terrestrial runoff or contaminants, which could lower immunity and increase disease impacts [65–67]. Additionally, San Francisco Bay historically experiences varying salinities and water outflow [68], abiotic stressors which may exacerbate disease impacts [69–71]. These conditions may have become more variable in recent years, as changing climate has been associated with higher temperatures and torrential rains [72, 73], possibly leading to an increase in the frequency and severity of disease events [74, 75]. Both warmer [2, 11] and colder [3] temperatures have been associated with SSWD, and further study is needed to elucidate these effects. Little attention has been given to the effects of salinity on SSWD, though results of a laboratory challenge experiment suggest correlation between SSWD and lower salinity in *Leptasterias* [13]. Sea stars are often considered stenohaline organisms that have limited osmoregulatory abilities [76, 77], and although some evidence for local adaptation to different salinities exists (e.g., [69, 77]), it is unclear how these varying environmental conditions will affect intertidal organisms’ immunity and reactions to disease events. Hence, the significance of local variation in environmental stressors cannot be ignored.

### Comparison to disease signs in other species

Comparing disease-affected organisms with different life histories remains a crucial goal of ecological surveillance. In both field and laboratory settings, morphological signs associated with SSWD appeared similar to those observed in larger asteroids, including white lesions on the rays, twisting arms, and loss of rays [2, 3, 6, 35]. When initially observed, similarity in physical disease signs across species led researchers to conclude that SSWD represented a single disease affecting multiple species across sites and regions that could be traced to a specific pathogen [6]. More recent studies of SSWD, however, suggest a syndrome caused by several interacting factors that may have variable impacts across species [12], suggesting further molecular work is needed to elucidate the underlying mechanism of SSWD and determine whether this mechanism is present across species.

### Challenges of documenting SSWD in *Leptasterias*

We encountered several challenges when observing physical signs of SSWD in *Leptasterias* in the field. First, their small size relative to more commonly studied stars [78] may cause them to be washed away in later stages of disease, when they begin to “melt” and lose their ability to remain attached to the substrate, biasing collection towards less diseased individuals. Lack of severely diseased individuals may also complicate comparison to larger asteroid species that are easier to find, such as *P*. *ochraceus*, both by leading researchers to underestimate the number of *Leptasterias* present at a given site and by preventing adequate sampling and observation of disease signs in individual sea stars.

Second, *Leptasterias’* often mottled coloration complicated our assessment of physical damage. Many individuals show speckled, grainy patterns, often with patches of white and light-grey (e.g. Fig 3B and 3G). These features resemble the lesions associated with SSWD (Fig 3C–3G, [2, 35]) and so led to some ambiguity when assessing sea stars that were less severely affected. To combat these challenges, multiple researchers assessed each sea star so that a consensus could be reached, microscopes were used to scrutinize each sea star in great detail, and comparison was made to stars that showed disease signs in the lab.

Third, the physical effects of SSWD in *Leptasterias* remain speculative. We based identification of disease signs in *Leptasterias* on signs reported in other species, such as *P*. *ochraceus* [2, 3, 35], due to the observed similarities of physical SSWD impacts across species. However, as a common cause for SSWD-related mortality across species remains elusive [12], we cannot conclude with certainty that the physical signs are the same. More sampling of diseased individuals, further comparison between disease signs across affected species, and molecular analysis are necessary to elucidate the physical effects of SSWD.

### Implications of *Leptasterias* population decline

We report a pattern of local absence in timed count surveys, in at least 5 populations of *Leptasterias* located in the outflow shadow of San Francisco Bay (Fig 2). This represents a decrease in abundance and potentially may also represent local extirpations of populations in a clade-specific pattern, which may have far-reaching ecological implications. *Leptasterias* are ecologically important intertidal predators, exerting top-down effects on the abundance and behavior of prey species such as the snail *Tegula funebralis* through density- and trait-mediated indirect interactions [22, 79, 80]. Recently, *Leptasterias* removal was correlated with increased *T*. *funebralis* density, leading to increased density of unpalatable algal species over a ten month period [81], suggesting that changes in community composition may be rapid and severe. *Leptasterias* are also competitors with larger asteroids such as *P*. *ochraceus* [22, 79, 80], though are thought to be competitively inferior “mesopredators” [79]. Although the observed reduction of apex predators such as *P*. *ochraceus* [2, 3, 7, 10, 11] could open up more resources to *Leptasterias*, this mesopredator release [82–85] may be offset by proportional declines in *Leptasterias* populations associated with SSWD, as well as differences in life history that make it harder for *Leptasterias* populations to recover. Predicting long-term ecological impacts of SSWD is challenging due to the variable impacts of SSWD across species and locations [2–4, 6], and future studies should continue to document and compare population declines across species.

### Effect of MgCl_2_ on SSWD disease progression

Laboratory experiments and observations conducted on *Leptasterias* spp. indicate that SSWD may not inevitably lead to death in this species. Affected *Leptasterias* spp. experienced a slowed or reduced disease progression after exposure to a combined treatment of MgCl_2_ and ice. The current mechanism by which MgCl_2_ affects sea stars still remains unknown; however, these results suggest MgCl_2_ may slow or halt either primary or secondary disease progression in aquarium settings. It may play a role in eliminating harmful secondary bacterial infections, allowing sea stars to better cope with viral loads [86–88]. Oysters exposed to MgCl_2_ saw an inhibition in phenoloxidase activity, which abated after 96 hours [89]. The phenoloxidase pathway is associated with the enveloping of foreign material in the haemolymph of invertebrates, and the production of antibacterial products [90–93]; thus, it seems counterintuitive that inhibition of phenoloxidase defenses would slow disease progression. MgCl_2_ alters the ionic environment of tissues, thereby affecting the mechanical properties of tissues and making it a useful tool for invertebrate anesthetization [94]. Comparison of the microbiomes of treated and untreated sea stars may reveal insights into the effect of magnesium chloride and is currently being investigated. Additionally, a more extensive and detailed study with precise measures of lesion size could elucidate the effect of MgCl_2_ on sea stars afflicted with SSWD.

## Conclusions

Due to sea stars’ important predatory role in rocky intertidal ecosystems, it is critical to determine how populations may recover from mortality events, and we must explore treatment options when possible. Data on both brooding and broadcast spawning asteroids will facilitate improved understanding of disease dynamics of SSWD, and given the severity and widespread effects of this disease (both in space and time), it is critical to establish pre-disease baselines to determine population-level impacts. The use of shared datasets and data repositories, such as seastarwasting.org, coupled with continued monitoring will help further our understanding of SSWD and its population dynamics. Further, characterizing pathology of SSWD is essential to understanding dynamics and impacts. Field surveys supplemented by careful documentation of disease progression in the lab are central to this process. Finally, we show that magnesium chloride treatment may slow or halt SSWD progression of sea stars in aquaria, and its role in infection should be investigated further.

## Supporting information

S1 Table Site names and locations. “Map color” correspond to colored regions in Fig 1. “*Leptasterias* counted” and “abundance rank” columns refers to combined data (see methods). “*Leptasterias*/observer hour” column refers to Cohen Lab timed counts. “Observer hours” column refers to Cohen Lab timed counts. “Data used in Fig 2” column indicates whether data were included in Fig 2, our heatmap. “Previous die-off observed [32]” column indicates whether population declines were reported in *Leptasterias* during a HAB event in [32]. “Source” column indicates where data came from: Cohen lab timed counts (“Cohen TC”), Cohen lab data not exclusively collected for population abundance monitoring (“Cohen other”), the MARINe online repository [35]: https://www.eeb.ucsc.edu/pacificrockyintertidal/data-products/sea-star-wasting/ (“MARINe”), or from [32] (Jurgens et al.).
(XLSX)

S2 Table Sites used for disease sign observation and photography. “Map color” correspond to colored regions in Fig 1.
(XLSX)
